# Antibacterial Effects of Modified Implant Abutment Surfaces for the Prevention of Peri-Implantitis—A Systematic Review

**DOI:** 10.3390/antibiotics10111350

**Published:** 2021-11-05

**Authors:** Marie-Elise Jennes, Michael Naumann, Simon Peroz, Florian Beuer, Franziska Schmidt

**Affiliations:** Charité—Universitätsmedizin Berlin, Corporate Member of Freie Universität Berlin and Humboldt Universität zu Berlin, Department of Prosthodontics, Geriatric Dentistry and Craniomandibular Disorders, Assmans-hauser Straße 4-6, 14197 Berlin, Germany; michael.naumann@charite.de (M.N.); simon.peroz@charite.de (S.P.); florian.beuer@charite.de (F.B.); franziska.schmidt2@charite.de (F.S.)

**Keywords:** peri-implantitis, prevention, implant, abutment, antibacterial coating

## Abstract

The aim of the present study was to systematically review studies investigating antibacterial implant abutment surfaces or coatings, which may suppress bacterial growth to prevent plaque-induced peri-implant inflammatory disease. Data were collected after identification of case, assay/laboratory procedure, predicate/reference standard and outcome (CAPO). Seven hundred and twenty (720) records were identified through data base searching. After screening nine publications fulfilled inclusion criteria and were included. The following surfaces/coatings showed antibacterial properties: Electrochemical surface modification of titanium by the anodic spark deposition technique; doxycycline coating by cathodic polarization; silver coating by DC plasma sputter; titanium nitride; zirconium nitride and microwave assistant nano silver coating. Since the current state of the literature is rather descriptive, a meta-analysis was not performed. While several abutment coatings showed to have antibacterial capacity, some of them also influenced the behavior of investigated human cells. None of the studies investigated the long-term effect of surface modifications. Since surface changes are the main contributing factor in the development of antibacterial effects, the biodegradation behavior must be characterized to understand its durability. To date there is no effective structure, material or strategy to avoid peri-implant inflammation used as clinical routine. Furthermore, clinical studies are scarce.

## 1. Introduction:

With a prevalence of 22% [1] peri-implant inflammation represents one of the most frequent complications in dental implantology affecting both the surrounding soft and hard tissues, which can lead to implant loss [2].

The risks for the development of peri-implant inflammation have been widely studied and were summarized in the Consensus Report of the Sixth European Workshop on Periodontology. Poor oral hygiene, a history of periodontitis and smoking were associated with peri-implant disease [3]. Once peri-implantitis is established, it does not respond predictably to treatment. Therefore, it appears that the best management of plaque-induced peri-implant inflammatory diseases is prevention [4]. To avoid the development of peri-implantitis not only solid osseointegration of the implants but also a robust soft-tissue integration at the transmucosal region is mandatory [5], since it is the first barrier against a bacterial invasion. It consists out of a sulcular epithelium, junctional epithelium and fibrous connective tissue between the epithelium and the first bone-to-implant contact [6]. 

In general peri-implant inflammation can be divided into mucositis and peri-implantitis. Mucositis describes a bacteria-induced, reversible inflammatory process of the peri-implant soft tissue with reddening, swelling and bleeding on periodontal probing. [4] In contrast, peri-implantitis is an extension of peri-implant mucositis, which is characterized by the presence of bone loss (≥ 2 mm) [7], by increased probing depth and bleeding and/or suppuration on probing [4]. The pathological changes of the tissues are caused by a bacterial invasion, which already occurs within 30 min after surgery resulting in inflammatory cell response [8,9]. Therefore, preventing bacterial invasion already within implant surgery for example by applying antibacterial-coated cover screws might be helpful, however, they are not on the market yet. Coatings for both implants and abutments have already been developed but are not yet clinical routine [10,11,12,13,14,15,16,17,18]. These surface modifications can be achieved through different surface treatments and allow a reproducible control of required surface properties on nearly every part of an implant [19].

The objectives of the present study are to review and evaluate material, structures or strategies to prevent plaque-induced peri-implant inflammatory disease, i.e., peri-implantitis, by means of antibacterial implant abutment surfaces or coatings for their antibacterial properties suppressing bacterial growth. 

## 2. Material and Methods:

This systematic review was performed adhering to Transparent Reporting of Systematic Review (PRISMA 2020) guidelines [20]. Pre-registration was not performed. Before starting the systematic literature research, the CAPO question [21] was stated, as it can be used to evaluate studies in evidence-based laboratory medicine. 

CAPO question:

C (Case): Prevention of peri-implantitis through antibacterial implant-abutment coatings
A (Assay or laboratory procedure): analyzing antibacterial activity and cytotoxicity
P (Predicate/reference standard): abutment material without coating
O (Outcome): cell viability, bacterial death

The resulting question was the following:

Do abutment coatings suppress bacterial growth or interfere with human cells in comparison to uncoated implant abutments?

### 2.1. Eligibility Criteria

The following criteria were used to include or exclude published articles.

### 2.2. Information Sources and Search

#### 2.2.1. Electronic Search

The electronic databases PubMed and Cochrane Central Register of Controlled Trials were searched on 23 April 2021 for studies published until April 2021. The specific search protocol can be seen below:

#### 2.2.2. Search Strategy

##### PubMed

(((antibacterial) AND (surface)) OR (coating) AND (abutment)).

##### Cochrane Central Register of Controlled Trials

(antibacterial): ti,ab,kw AND (surface): ti,ab,kw OR (coating): ti,ab,kw AND (abutment): ti,ab,kw

Two reviewers (M.-E.J., F.S.) independently screened titles and abstracts of studies according to the predefined search strategy and additional sources. Following this, selected full-text articles were again reviewed and discussed by the two reviewers (M.-E.J., F.S.). The kappa value was calculated as a measure of agreement between the readers after screening the full texts and any disagreements were clarified by discussion with a third reviewer (M.N.). Articles found from additional sources as hand search and grey literature were also screened following the same systematic procedure. The following data were extracted from the included studies by two authors independently (M.-E.J., F.S.): study design, type of coating, control group, test procedure, influence of abutment coating on cell behavior, influence of abutment coating on bacteria, missing or unclear information. EndNote (Version 20, Clarivate Analytics, London, UK) was used as the software to organize and screen the extracted articles.

### 2.3. Risk of Bias Assessment

The included clinical studies were assessed by the revised RoB tool based on the Cochrane RoB assessment method by Higgins et al., (2016) [22]. Preclinical animal studies were assessed by a tool developed by the Systematic Review Centre for Laboratory animal Experimentation (SYRCLE). This RoB tool is based on the Cochrane tool, updated for specific biases related to animal studies as published by Hooijmans et al. [23].

For in vitro studies, also known as mechanistic studies, there are no established RoB tools comparable to the ones for clinical and animal studies. Therefor the Office of Health Assessment and Translation (OHAT) of the U.S. Department of Health and Human Services developed a tool to assess the RoB assessment of in vitro studies. Details can be found in the “Handbook for Conducting a Literature-Based Health Assessment Using OHAT Approach for Systematic Review and Evidence Integration” [24].

## 3. Results

### 3.1. Study Selection

**Figure 1:**
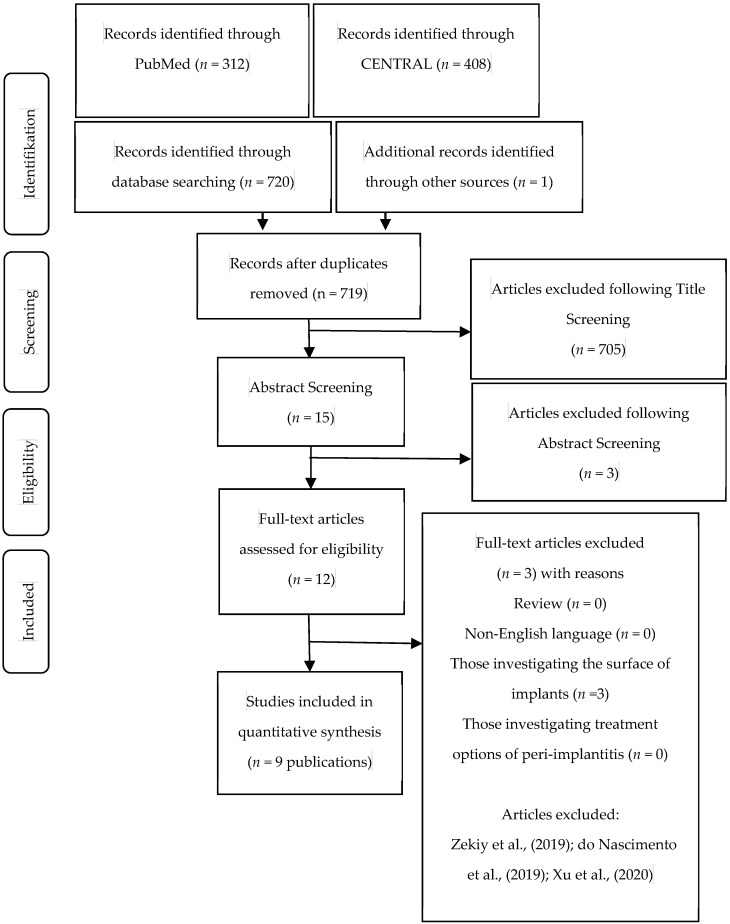
Data extraction process and study selection.

The data extraction process and the study selection can be seen in Figure 1. The initial search yielded 720 articles. It was reduced to 719 articles by removing duplicates. One article was identified additionally through hand search. After title screening 705 articles were excluded, which resulted in a number of 15 potentially eligible articles. After reviewing the abstracts the number of eligible articles was reduced to 12. A final review resulted in the selection of nine articles [10,11,12,13,14,15,16,17,18]. For the assessment of inter-reviewer agreement the *k* value was calculated and showed a value of *k* = 1. The extracted data of included studies can be seen in Table 1. Since the current state of the literature is rather descriptive, it was not possible to perform further statistical evaluations. 

### 3.2. Risk of Bias Assessment (RoB)

**Figure 2:**
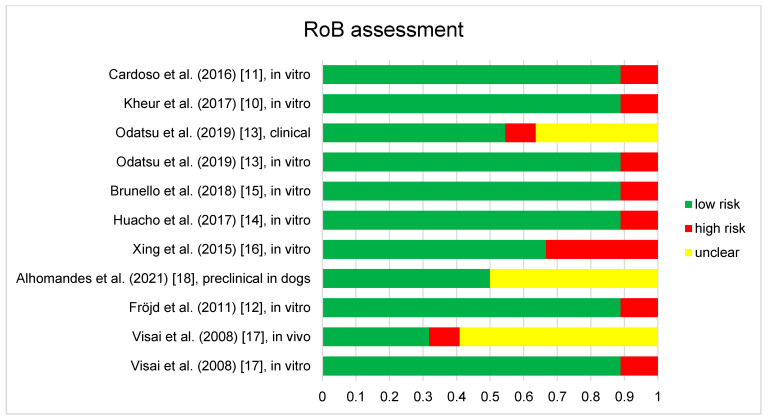
Results of the risk of bias assessment of the 9 studies included in this review. Studies containing different aspects, such as in vitro and clinical parts are assessed separately and displayed as such (e.g., Odatsu et al., (2019) [13], clinical and Odatsu et al., (2019), in vitro [13]), as different RoB tools were employed.

The results of RoB assessments are included in Figure 2. Any disagreements were clarified by discussion with the third reviewer (M.N.). Eight of the discussed publications contained in-vitro studies. They generally showed low risk of bias for sample randomization, allocation concealment and experimental conditions. Samples were randomized and homogenous cell and bacterial suspension were employed with unified experimental conditions independent of control or test samples. The only exception was Xing et al., (2015) [16], who specified that they selected samples for in-vitro testing, which were considered representative. However, in our opinion this does not comply with an adequate randomization of samples and culture. Further, all discussed in-vitro studies did not describe blinding during the study, such as employing robotic or automated systems, which was considered high bias risk. Other bias factors, such as incomplete data, exposure characterization, outcome assessment, reporting and other were uniformly considered as low risk of bias. 

Two clinical studies [13,17] were found to be under risk of bias. They were assessed according to bias arising from the randomization process, bias due to deviations from intended interventions, bias due to missing outcome data, bias in measurement of the outcome and bias in selection of the reported result [22]. Both studies showed generally low risk of bias in all areas. Specifics can be found in the Figure 2. Visai et al., [17] did not employ an appropriate analysis to estimate the effect of assignment to intervention. Furthermore, they showed no evidence that the result was not biased by missing outcome data, which was considered high risk of bias. Odatsu et al., [13] did not show evidence that the result was not biased by missing outcome data and did not clearly state whether assessors were aware of the intervention received by study participants, which was also considered as high risk of bias. 

Finally for the preclinical study of Almohandes et al., [18] selection bias was found to be low risk or not applicable, performance bias was found to be low risk or not applicable, detection bias was found not applicable, attrition bias, reporting and other bias were found to be low risk. Bias considerations found not applicable were due to the low number of animals and the study design, as all animals received test and control group samples.

### 3.3. Study Characteristics

The extracted data (study design, type of coating, control group, test procedure, influence of abutment coating on cell behavior, influence of abutment coating on bacteria, missing or unclear information) of the included studies can be seen in Table 2.

#### 3.3.1. Abutment Surface Modification

The included studies investigated the antibacterial properties of different modified implant abutment surfaces. Eight studies modified titanium and one study titanium zirconium samples. Furthermore, eight studies [10,11,12,13,14,15,17,18] evaluated the antibacterial properties of inorganic abutment coatings. Only one study [16] examined an organic coating. All coatings were firmly bonded to the abutment surface either chemically or mechanically. In total six different surface modification methods were performed (anodic oxidation, cathodic polarization, sol-gel treatment, physical vapor deposition (PVD), heat treatment and microwave assistant coating). One study [14] did not specify the method of surface modification. Anodic oxidation is an electrochemical process for the production of an oxide layer on metallic substrates. In this process, an electrical bias voltage with comparatively low currents is applied while the substrates are immersed in an acid bath [25]. In this context anodic spark deposition technique (ASD) performed by Visai et al., [17] is a novel anodic oxidation technique to integrate calcium and phosphate ions within the microporous structure of titanium oxide [26]. In addition, a thickening of the oxide layer and a change in titanium color can be achieved for aesthetic purposes [17]. Anodic oxidation was also performed by Fjörd et al., [12] and Brunello et al. [15]. Cathodic polarization is described as an alternative electrochemical treatment, which can cause a change in surface roughness and the deposition of biomolecules under lower temperatures [27]. Since the binding of biomolecules to native TiO_2_ layers of titanium has proven to be difficult due to the low reactivity of this surface, cathodic reduction in acidic solutions can be used to create a hydrogen-rich surface on which biomolecules can bind more efficiently [28].

Sol-gel treatment is a wet-chemical technique, which is primarily used for the preparation of metal oxides. It is based on a chemical solution that functions as a precursor for an integrated network (or gel) of discrete particles or network polymers [29]. In this context sol-gel derived nanoporous titanium oxide should enhance soft tissue attachment [12]. 

PVD refers to a range of vacuum deposition processes that can be used to create thin films and coatings. It is characterized by a process in which the material passes from a condensed phase to a vapor phase and then back to a condensed thin film phase [30].

The microwave assisted coating for the production of nanoparticles, performed by Odatsu el al., (2020) {Odatsu, 2020 #682}, is a technique that provides better technical control over the separation of nucleation and growth stages in the synthesis of nanoparticles when the reaction is started at room temperature [30]. Another possibility for the formation of nanoaggregates is heat treatment [13].

#### 3.3.2. Control Groups

With the exception of one study [16] all included studies used titanium as control. Only Xing et al., [16] used titanium-zirconium samples as control. 

#### 3.3.3. Antibacterial Properties of Investigated Implant Abutment Surfaces

##### In Vitro Studies

Titanium oxide (TiO_2_) coating and calcium-treated surfaces did not influence biofilm formation of *Streptococcus sanguinis* and *Actinomyces naeslundii* after an incubation period of 2 h and 14 h. [12]. Cardoso et al., [11] investigated diamond-like carbon films (DLC) with and without embedded silver nanoparticles to prevent bacterial leakage through internal and external hexagonal implants. Although the DLC film reduced the absolute percentage of leakage after five days, there were no statistically significant differences between the two types of implants. Huacho et al., [14] also examined the antibacterial properties of DLC in *Escherichia coli* and showed that DLC has no antimicrobial properties and does not interfere with bacterial adhesion after 3 h and 24 h.

Electrochemical surface modification of titanium by anodic spark deposition (ASD), performed in a calcium phosphate enriched solution, showed statistically significant higher antibacterial activity in ASD samples compared to titanium. Also a statistically significant reduction in bacterial attachment was observed after an incubation period of 3 h and 24 h [17]. Etched titanium disks coated with silver using a DC plasma sputter coating instrument showed a significant reduction of viable counts of *Staphylococcus aureus*, *Streptococcus mutans* and *Pseudomonas aeruginosa* after 6 h of incubation. In this context, the time of plasma sputter coating influenced the antibacterial properties [10]. Titanium disks coated with titanium nitride or with zirconium nitride by anodization showed a higher percentage of dead bacteria in the biofilms in comparison to uncoated titanium disks, when evaluating the biofilm growth of *Streptococcus salivarius*, *S. sanguinis*, *S. mutans*, *S. sobrinus*, and *S. oralis* [15]. The number of colony-forming units of *Staphylococcus aureus* on titanium disks coated with microwave assistant nanosilver were suppressed significantly in comparison to pure titanium after an incubation period of 120 h [13].

Xing et al., [16] examined an organic abutment coating. They coated titanium-zirconium (machined or machined and acid etched) samples with doxycycline by cathodic polarization and found an initial bacteriostatic property from the burst release of doxycycline within the first 24 h, and a longer term antibacterial potential for at least 2 weeks. 

##### In Vivo Studies

Visai et al., [17] and Odatsu et al., [13] conducted in-vivo studies in addition to the in-vitro studies previously described. Visai et al., [17] did not perform their in vivo study on implant abutments, but rather in silicone appliances, where 5 mm wide titanium disks were fixed mechanically on the buccal sides of appliances. The volunteers had to wear the appliances, consecutively for 24 h removing them only for meals without performing any oral hygiene procedure. After 24 h the density of bacteria on the appliances was examined. The amount of biofilm formation was lower in the non-treated titanium group, but without statistically significant difference.

Odatsu et al., [13] included 19 patients and compared uncoated with coated implant abutments on two distal implants of each patient to investigate the area of plaque coverage. After 28 days, all abutments were analyzed by plaque staining. Nano silver coated titanium abutments showed significantly smaller areas of plaque accumulation in comparison to the uncoated control group. 

Furthermore Almohandes et al., [18] performed a preclinical in-vivo study in dogs and showed that titanium-bismuth-gallium coating did not prevent biofilm formation on implant abutments 6 and 7.5 month after placing ligatures around the implant abutment for 4 weeks.

#### 3.3.4. Influence of Implant Abutment Coating on the Behavior of Examined Cells

Besides investigating antibacterial properties of implant-abutment coatings Visai et al., [17], Almohandes [18], Huacho et al., [14], Kheur et al. [10], Brunello et al. [15] and Odatsu et al. [13] also examined the influence of abutment coatings on cell behavior or on peri-implant tissues. 

The metabolic activity of MG63 cells was not influenced by electro-chemical surface modification of titanium by ASD, performed in a calcium phosphate enriched solution. Furthermore, the cell number of MG63 cells on ASD treated samples was statistically significantly higher compared to the negative control [17].

In the study of Almohandes et al., [18] biopsies were obtained eight months after abutment connection and prepared for histological analysis. The investigated Ti-Bi-Ga coating did not influence the host response in the adjacent peri-implant mucosa.

Human gingival fibroblasts (HGF) incubated directly on sample disks (titanium samples coated with titanium nitride, or with zirconium nitride) did not show differences in proliferation. Morphological analysis with scanning electron microscopy, hemolysis test, Ames test, ribonucleic acid (RNA) extraction, first-strand complementary deoxyribonucleic acid (cDNA) synthesis, Real-time polymerase chain reaction (PCR) and indirect immunofluorescence was performed. The genes considered were talin, alpha-actinin, vinculin, zyxin, paxillin, vitronectin, focal adhesion kinase (FAK), and collagen type I, all involved in cell adhesion. Good mRNA relative expression levels were found on all the surfaces examined, but the highest gene expression values were observed on the ZrN-treated disks. HGF adhered on all disks and did not show differences in vinculin expression. None of the samples were hemolytic and no mutagenic activity was revealed for any of the surfaces tested [15]. The influence of microwave-assistant nano silver coating on pure titanium on actin filaments of HGF was investigated by Odatsu et al., [13] with immunofluorescence microscopy. The number of attached cells and cell proliferation was examined through MTS assay. The results did not show statistically significant differences between control and nano silver coating regarding cell number, cell shape and proliferation. Etched and non-etched titanium disks coated with silver using a DC plasma sputter coating instrument were analyzed regarding their cytotoxicity by MTT assay. The cell morphology was analyzed by phase contrast microscopy. In the study of Kheur et al., [10], etched titanium disks coated with Ag showed 40% less and unetched titanium disks coated with Ag 20% less cell viability of HGF in comparison to the control. Phase contrast images showed in all samples rounding and clumping of cells compared to the flat and elongated cells of the control group [10]. When investigating the influence of DLC on HaCat cells, Huacho et al., [14] performed MTT-assays and found the coatings to be mildly cytotoxic.

## 4. Discussion

The objectives of this systematic review were to summarize and evaluate the state of research on antibacterial surfaces or coatings on implant abutments as a primary prevention against mucositis and peri-implantitis. Therefore, a systematic search strategy was applied for the databases PubMed and Cochrane Central Register of Controlled Trials. The search resulted in nine included studies (seven in vitro; two in vivo). We found that several abutment coatings investigated in the included studies suppressed bacterial growth or influenced the behavior of investigated human cells, indicating less cell viability or mild cytotoxicity.

The PICO question is easily applicable for the evaluation of most therapeutic or interventional studies but can be inappropriate for use in evidence-based laboratory medicine. Therefor the CAPO question was proposed by Christenson [21]. As there is no consensus on an appropriate tool for the risk of bias assessment of in-vitro studies [31] the tool developed by the United States national toxicology program was applied [32]. 

As limitations of this study we can mention that only a few studies were included in the systematic review and none of the studies investigated the long-term effect of implant abutment coatings. All studies investigated antibacterial properties with different bacteria and laboratory methods. Likewise, the cells used differed in the included studies, preventing meta-analysis for outcomes. Furthermore, only two clinical studies and one pre-clinical study in dogs were performed. It is also known that studies presenting significant results are time more likely to be published compared to null results. This so-called publication bias may also have influenced our findings [33].

Five of the included studies showed antibacterial properties of investigated implant abutment surfaces/coatings. Electrochemical surface modification of titanium by ASD, performed in a calcium phosphate enriched solution showed statistically significant less bacterial attachment and a lower amount of biofilm formation on the treated than on the non-treated surfaces. During ASD, the titanium oxide film is transformed into the crystal structure of anastasis and thus exhibits photocatalytic activity [17]. However, no additional photocatalytic activation of bacterial colonized surfaces was mentioned in the study of Visai et al. [17], which is described as the attributing factor of antibacterial properties of titanium oxide surfaces [34]. Likewise Fröjd et al. [12] investigated TiO_2_ coating by sol gel treatment and did not describe any antibacterial effect. The investigated surface was not irradiated with UV light in this study either. As the antibacterial activity of TiO_2_ by UV-Light exposure has been confirmed in other studies [35,36,37], the use of TiO_2_ surfaces without additional photocatalytic activation appears to have no influence on bacterial colonization of implant components.

Titanium-bismuth-gallium coating did not prevent biofilm formation in a preclinical in-vivo study with dogs. The combination of titanium, bismuth and gallium in terms of bactericidal capacity was to the best of our knowledge not investigated before, although bismuth compounds showed antibacterial properties against *Aggregatibacter actinomycetemcomitans*, *Streptococcus mutans*, methicillin-resistant *Staphylococcus aureus* [38], *Actinomyces naeslundii* and *Streptococcus sanguinis* [39].

Despite the investigations of Xing et al., the antibacterial effects of doxycycline abutment coatings have not been described in the literature yet. In general, the antibacterial effect of doxycycline is in accordance with the recent literature and shows, when applied systematically, steady-state levels in gingival crevicular fluid [40,41,42]. However the local application of doxycycline as a treatment for periodontitis is discussed controversially [43,44,45,46], and the current available scientific information on the use of locally or systemically administered antibiotics is insufficient to allow any firm specific recommendations for their use [47].

The biomedical application of DLC films can promote the growth of cells like fibroblasts, osteoblasts, and macrophages, without signs of inflammation or cytotoxicity [48]. In general the primary bactericidal mechanism of carbon based materials is the irreversible damage of the outer membrane of bacteria and a following release of intracellular content [49]. Although antibacterial properties of DLC films were shown [36,49], the investigated DLC-coatings of the included studies [11,14] did not show antibacterial capacity in comparison to titanium. In this context no difference in the adhesion of bacteria in comparison to stainless steel were stated [50]. These inconsistent results could be due to the use of different bacteria, when testing antibacterial properties of the respective materials.

Silver nanoparticles have been shown to be effective biocides against bacteria, fungi and viruses [51,52,53,54,55]. This makes silver-coated surfaces interesting for dental applications. The antibacterial effect of silver nanoparticles has not been fully elucidated yet. The currently accepted theory on their antibacterial effect describes an uptake of free silver ions followed by a disturbance of ATP production and DNA replication, a generation of ROS by silver nanoparticles and silver ions and a direct damage of cell membranes by silver nanoparticles [56]. Many studies can be found in the literature investigating the influence of silver nanoparticles in dental materials for prosthetic, restorative, endodontic, orthodontic, periodontal and implant treatment and show promising results regarding their antibacterial properties [55]. However, these studies are mostly in-vitro studies. Clinical studies are crucial to further evaluate these effects in different dental applications. 

The antibacterial capacity of titanium nitride and zirconium nitride described in the paper of Brunello et al., has already been reported several times in the literature [19,57,58,59]. Furthermore a change in microbial community composition was demonstrated on titanium surfaces modified with ZrN coating, which might help to influence the adhesion of less pathogenic bacteria and thereby reducing the risk of peri-implantitis [60]. An antibacterial effect has already been demonstrated in clinical practice with in-vivo studies [57,59]. However, these trials were conducted in an abstract clinical set-up with splints where modified materials were embedded. Therefore, further clinical studies are needed to investigate the effect of these surfaces in their actual clinical use for the prevention of peri-implantitis. 

When discussing the antibacterial effect of coated surfaces, the duration of the effect should be of particular interest. Early bacterial contamination can lead to bone loss even before incorporating prosthetic restoration, which may finally result in implant loss, i.e., early implant failure. On the other hand, implants with good bone-to-implant contact might show a loss of the marginal bone over the service time of the prosthetic restoration and can thus be assigned to a late implant failure [61]. Therefore, a desirable effect of antibacterial coatings should be both short and long term. Unfortunately, the duration of the effect is usually not examined in most of the studies and none of the included studies investigated the long-term effect and the corrosion behavior of modified surfaces. Since surface changes are the main contributing factor in the development of antibacterial effects, the biodegradation behavior must be precisely characterized to understand its durability [62], and has to be part of future investigations.

The need for the prevention of peri-implant inflammations is reflected in its prevalence, which was reported to be 43% for mucositis and 22% for peri-implantitis in a recently published systematic review [1], and in the rapid, i.e., within minutes, bacterial colonization of implant components after implant placement [8]. But as the outcome of peri-implantitis therapy is still considered unpredictable [3], it seems of particular interest to focus on prevention by avoiding bacterial colonization right after implant placement. Future research should focus on antibacterial surfaces that inhibit the growth of certain bacteria such as *Porphyromonas gingivalis*, *Staphylococcus aureus*, *Staphylococcus anaerobius*, *Streptococcus intermedius*, *Streptococcous mitis*, *Tanerella forsythia* and *Treponema socranskii*. Bacterial colonization with these bacteria has been shown to be four times higher on peri-implant sites than on healthy implants [9]. Besides the antibacterial effect itself, the antibacterial effect over a longer period of time, i.e., years of clinical use, should also be evaluated. In addition to the surface modification of implant abutments described above, the use of bioactive ions such as Zn^2+^ appear to be promising [63,64,65,66,67]. Their impact on bacterial growth in the field of implant dentistry should be investigated using antibacterial modified closure screws, healing caps and restorative components as implant abutments or crown material.

## 5. Conclusions

The CAPO question can be answered as followed: Several abutment coatings investigated in the included studies suppressed bacterial growth (electrochemical surface modification of titanium by the anodic spark deposition technique; doxycycline coating by cathodic polarization; silver coating by DC plasma sputter; titanium nitride; zirconium nitride and microwave assistant nano silver coating). Furthermore, abutment coatings of diamond-like-carbon and silver coating by DC plasma sputter influenced the behavior of investigated human cells, indicating less cell viability or mild cytotoxicity. Since clinical studies are scarce, further clinical investigations need to be conducted.

## Figures and Tables

**Table 1:** Inclusion and exclusion criteria.

Inclusion criteria	Clinical studies and case series with at least five participants Animal studies In-vitro studies in peer-reviewed journals With the focus on prevention of peri-implantitis Through antibacterial abutment coatings Articles published in English or German language
Exclusion criteria	Reviews Those only investigating the surface of implants Those investigating treatment options for peri-implantitis Published articles that were not available in English or German language

**Table 2:** Study selection and extracted data from the included studies.

Author (Year) [10,11,12,13,14,15,16,17,18]	Study Design	Abutment Coating	Control Group	Cells Used	Bacteria Used	Test Procedure	Influence of Abutment Coating on Cell Behaviour	Influence of Abutment Coating on Bacteria	Missing or Unclear Information
Visai et al., (2008) [17]	In vitro and in vivo	Electrochemical surface modification of titanium by Anodic Spark Deposition technique (ASD), performed in a calcium phosphate enriched solution	Grade 2 titanium	MG63—human osteosarcoma cell lines and L929—murine fibroblasts	*Streptococcus mutans*, *Streptococcus salivarius* and *Streptococcus sanguis*	Surface microstrucutral characterization; in vitro metabloic cell activity with Alamar blue Solution; Bacterial adhesion and growth inhibition by couting Colony Forming Units/cm^2^; in vivo: customized appliances with samples on 8 volunteers measuring bacteria density after 24 h with SEM	Metabolic activity showed no differences between the groups; Cell number of MG63 cells on ASD treated sample statistically significant higher compared to negative control	After 24 hr of incubation, the results of antibacterial activity were greater on AB than on Ti samples (*p* < 0.05) following this trend: *S. salivarius* > *S. sanguis* > *S. mutans*; After 3 hr of incubation, bacterial attachment was consistently reduced on ASD samples than on Ti samples for all tested strains (*p* < 0.05). The amount of biofilm formation was lower, on average, in the non-treated titanium than in the ASD sample	-
Fröjd et al., (2011) [12]	In vitro	Sol-gel treatment to create a nanoporous TiO_2_ coat (SG), heat-treated in a similar way to the sol-gel treated discs (HT), or anodically oxidized and calcium treated (OC)	Grade 4 pure titanium	-	*Streptococcus sanguinis* and *Actinomyces naeslundii*	Assay for bacterial adhesion and early biofilm formation	-	No differences in the overall biofilm biovolume between the four surfaces were detected	-
Almohandes et al., (2021) [18]	Pre-clinical in vivo study in dogs	Titanium-bismuth-gallium (Ti-Bi-Ga) coating by physical vapour deposition (PVD)	Titanium Uni abutment (Astra Tech Implant SystemTM, Dentsply Implants IH AB)	-	Microbiological samples were collected and analyzed	2 months after implantation, ligatures were placed around implants and plaque formation was allowed until the end of the experimen; ligatures were removed after 4 weeks; Radiographs and microbiological samples were obtained from each implant site during the plaque formation period. Biopsies were obtained 8 months after abutment connection and prepared for histological analysis.	Ti-Bi-Ga coating did not influence the host response in the adjacent peri-implant mucosa.	Ti-Bi-Ga coating did not prevent biofilm formation	Titanium Ggade was missing. (According to Astra Tech Implant System; Dentsply Sirona: Ti6Al4V Grad 5)
Xing et al., (2015) [16]	In vitro	Doxycycline coating by cathodic polarization and polarization time of 1 h and 5 h and current density of 1 and 5 mA cm^−2^	Titanium–zirconium coin-shaped samples machined (M); and machined, acid-etched (MA) Control 1: cathodic polarization with Doxy at 1 mA cm^−2^ for 1 h; control 2: cathodic polarization with Doxy at 5 mA cm^−2^ for 3 h.	-	*Staphylococcus epidermidis*	Biofilm and planktonic growth assays using *Staphylococcus epidermidis*	-	Initial bacteriostatic property from the burst release of Doxy within 24 h, and a long-term antibacterial potential for at least 2 weeks. A higher amount of Doxy on the surface can be obtained by increasing polarization time from 1h to 5h and current density from 1 to 5 mA cm^−2^.	
Cardoso et al., (2016) [11]	In vitro	Diamond-like carbon (DLC) films (pure DLC and DLC with embedded silver nanoparticles [Ag-DLC]) were deposited on the abutment bases by plasma-enhanced chemical vapor deposition (PECVD)	Titanium abutments	-	*Enterococcus faecalis*	Indirect (or reverse) technique was used to assess bacterial leakage from internal and external hexagon implant-abutment connections; inner part of the implant was inoculated with 1 μL *E. faecalis* suspension (106 colony-forming units [CFU]/mL); After inoculation, the abutments were screw-retained to the implants with a torque of 20 Ncm. The quantity of inoculum was determined in a pilot study, in which the authors observed that >1 μL promoted leakage to the external side.	-	Percentage of bacterial leakage was 16.09% for EH implants and 80.71% for IH implants (*p* < 0.0001). Although the DLC film reduced the absolute percentage of leakage, there were no statistically significant differences between the two types of implants (*p* = 0.253 for EH implants and *p* = 0.535 for IH implants).	Abutment Material. The pictures show a titanium abutments but without specific caption
Huacho et al., (2017) [14]	In vitro	Diamond-like carbon (DLC)	Titanium	HaCat cells	*Escherichia coli*	Biocompatibility testing of DLC was performed by colorimetric analysis of methylthiazol tetrazolium (MTT); Bacterial Adhesion Test, Antimicrobial Test	Biocompatible, with mild cytotoxicity	DLC has no antimicrobial properties and does not interfere with bacterial adhesion when tested against *Escherichia coli*	The method for coating surfaces with DLC was not mentioned.
Kheur et al., (2017) [10]	In vitro	Etched and non-etched Ti discs were coated with silver using a DC plasma sputter coating instrument for 1, 2, 3 and 5 min	Grade 5 titanium discs	Human gingival fibroblast	*Staphylococcus aureus*, *Streptococcus mutans*, *Pseudomonas aeruginosa*	Bacterial adhesion assay; Cytotoxicity against HGF cells was assessed by MTT assay; Cell morphology by phase contrast microscopy	In comparison to the titanium control, a reduction (20%) in cell viability was observed insilver-deposited titanium abutment. For unetched-Ag coated samples; Ti-Ag(2), Ti-Ag(3) and Ti-Ag(5), although a reduction in viability was noted, the difference was not statistically significant. Similar reductions were noted in the case of etched Ag-coated samples; Ti-EAg(2), Ti-E-Ag(3). However, in a case of Ti-E-Ag(5), only 60% cell viability was observed; this decrease being statistically significant (*p* < 0.05) as compared to other time-points. Overall, at 72 h the toxicity of the specimens was in the order, Ti-E-Ag(5) > Ti-Ag(5) > Ti-EAg(3) > Ti-Ag(3) > Ti-E-Ag(2) > Ti-Ag(2)	In case of *S. mutans* and *P. aeruginosa*, the viable count reduced drastically after 6 h in all silver deposited abutments viz., Ti-Ag(1), Ti-Ag(2), Ti-Ag(3) and Ti- Ag(5). In the case of *S. aureus*, cells remained viable after contact with Ti-Ag(1) for 6 h. However, in a case of Ti-Ag(2), Ti-Ag(3) and Ti-Ag(5), the decrease in cell viability post 6 h contact was significant.	Antibacterial activity of etched titanium discs with silver coating was not mentioned.
Brunello et al., (2018) [15]	In vitro	Anodized, coated with titanium nitride, or coated with zirconium nitride. Anodization (Anodic oxidation) was performed with current of 2.2 A, voltage of 8 V in 5% phosphoric solution for 10 min. Coating was applied by PVD	Uncoated machined Ti disks	Human gingival fibroblasts	*Streptococcus salivarius*, *S. sanguinis*, *S. mutans*, *S. sobrinus*, and *S. oralis*	MTT Assay for proliferation analyzes of cells incubated directly on the discs; morphological analyzes with SEM; Hemolyses Test, Ames Test; RNA extraction and first-strand cDNA synthesis; Real-time PCR; Bacterial strains and biofilm quantification, indirect Immunfluorscence	No differences in proliferation between the samples. None of the samples were hemolytic; no mutagenic activity was revealed for any of the surfaces tested; The genes considered were talin, alpha-actinin, vinculin, zyxin, paxillin, vitronectin, focal adhesion kinase (FAK), and collagen type I, all involved in cell adhesion. Good mRNA relative expression levels were found on all the surfaces examined, but the highest gene expression values were observed on the ZrN-treated disks, HGF adhered on all disks; no difference in Vinculin expression;	The percentage of dead bacteria was higher in the biofilms grown on the TiN- and ZrN-coated disks than on the uncoated disks	-
Odatsu et al., (2020) [13]	In vitro and clinical study	Microwave assistant nanosilver coating on pure titanium	Pure titanium	Human gingival fibroblasts	*Staphylococcus aureus*	Immunfluorescence of actin filaments; number of attached cells, MTS assay for assessment of proliferation; Measuring colony forming units; Plaque covered area of abutments in vivo after 28 days	No statistically significant difference between control and nano-Ag coating regarding cell number and proliferation	Prevention of plaque accumulation by the nano-Ag coating.	-
